# Implications of NAD^+^ Metabolism in the Aging Retina and Retinal Degeneration

**DOI:** 10.1155/2020/2692794

**Published:** 2020-05-09

**Authors:** Ravirajsinh N. Jadeja, Menaka C. Thounaojam, Manuela Bartoli, Pamela M. Martin

**Affiliations:** ^1^Department of Biochemistry and Molecular Biology, Medical College of Georgia, Augusta University, Augusta, GA 30912, USA; ^2^Department of Ophthalmology, Medical College of Georgia, Augusta University, Augusta, GA 30912, USA; ^3^James and Jean Culver Vision Discovery Institute and Medical College of Georgia at Augusta University, Augusta, GA, USA

## Abstract

Nicotinamide adenine dinucleotide (NAD^+^) plays an important role in various key biological processes including energy metabolism, DNA repair, and gene expression. Accumulating clinical and experimental evidence highlights an age-dependent decline in NAD^+^ levels and its association with the development and progression of several age-related diseases. This supports the establishment of NAD^+^ as a critical regulator of aging and longevity and, relatedly, a promising therapeutic target to counter adverse events associated with the normal process of aging and/or the development and progression of age-related disease. Relative to the above, the metabolism of NAD^+^ has been the subject of numerous investigations in various cells, tissues, and organ systems; however, interestingly, studies of NAD^+^ metabolism in the retina and its relevance to the regulation of visual health and function are comparatively few. This is surprising given the critical causative impact of mitochondrial oxidative damage and bioenergetic crises on the development and progression of degenerative disease of the retina. Hence, the role of NAD^+^ in this tissue, normally and aging and/or disease, should not be ignored. Herein, we discuss important findings in the field of NAD^+^ metabolism, with particular emphasis on the importance of the NAD^+^ biosynthesizing enzyme NAMPT, the related metabolism of NAD^+^ in the retina, and the consequences of NAMPT and NAD^+^ deficiency or depletion in this tissue in aging and disease. We discuss also the implications of potential therapeutic strategies that augment NAD^+^ levels on the preservation of retinal health and function in the above conditions. The overarching goal of this review is to emphasize the importance of NAD^+^ metabolism in normal, aging, and/or diseased retina and, by so doing, highlight the necessity of additional clinical studies dedicated to evaluating the therapeutic utility of strategies that enhance NAD^+^ levels in improving vision.

## 1. Introduction

Nicotinamide adenine dinucleotide (NAD^+^) was discovered in 1906 as a coenzyme involved in yeast fermentation [1]. We now know it to be an important cofactor, required for at least 500 different enzymatic reactions in the body including those central to key metabolic pathways such as glycolysis, fatty acid (*β*) oxidation, the tricarboxylic acid (TCA) cycle, and oxidative phosphorylation as the redox interplay between the oxidized (i.e., NAD^+^) and reduced forms of NAD (i.e., NADH) governs the activity of critical enzymes in these pathways [2, 3]. NAD^+^ is also consumed in the processes of protein deacetylation and ADP-ribosylation by sirtuin and poly (ADP-ribose) polymerase (PARP), respectively [2, 4]. Further, the NAD glycohydrolases, CD38 and CD157 (BST1), consume NAD^+^ through the conversion of NAD into ADP-ribose (ADPR) or cyclic-ADPR [5]. Thus, the facilitation of biologic processes central to the maintenance of the living mammalian (e.g., metabolism, DNA repair, and gene expression) hinges upon the availability of NAD^+^. Congruent with its obligatory requirement in numerous extremely important biologic reactions, NAD^+^ levels in the average, healthy human adult are maintained relatively high, ~3 grams [6]! However, as age increases, NAD^+^ levels decline gradually. This age-related decrease in the availability of NAD^+^ has been linked strongly to processes relevant to normal aging and importantly also to the development and progression of a number of age-related diseases [6–8]. In 2000, Imai et al. made the groundbreaking discovery that yeast SIR2 (silent information regulator 2) and the mouse ortholog, SIRT1, transcriptional silencers and thereby regulators of longevity, are NAD^+^-dependent [9]. This discovery helped to explain mechanistically the link between impaired mitochondrial bioenergetics, reactive oxygen species generation, and aging and fueled new interest in understanding NAD^+^ biology and the importance of NAD^+^ metabolism in aging. It also renewed interest in the decades-old search for strategies to effectively augment NAD^+^ levels for therapeutic purposes.

The relationship between NAD^+^, health, and longevity though not completely understood undeniably exists. The current understanding of this subject has perhaps been most uniformly presented by Imai who in 2009 introduced the concept of the NAD World to explain the novel systemic regulatory network for metabolism and aging. Imai established that there are two critical components: (1) NAMPT- (nicotinamide phosphoribosyltransferase-) mediated systemic NAD biosynthesis as the pacemaker and driver of metabolism in tissues and organs and (2) the NAD-dependent deacetylase SIRT1 as the universal mediator of metabolic functions in various tissues [10]. He later revised the concept (NAD World 2.0), to better emphasize the importance of the intertissue communication between three key organs and tissues, namely, the hypothalamus, skeletal muscle, and adipose tissue which he referred to functionally as the controller, the effector, and the modulator, respectively [11]. With such rapid progress in this field, there have already been many excellent review articles about general NAD^+^ biology and its relevance to health and disease [4, 8, 12–16]. However, none has focused, as we do in the current review, on the specific importance of NAD^+^ metabolism in ocular diseases, an area that has received comparatively less attention.

## 2. NAD^+^ Synthesis in the Retina

**Figure 1:**
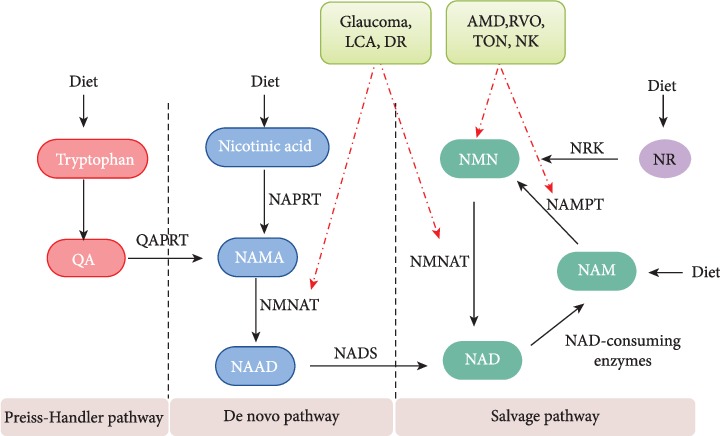
Overview of NAD^+^ biosynthesis pathways. NAD^+^ is mainly synthesized via the Preiss-Handler, *de novo*, and salvage pathways using tryptophan, nicotinic acid, and nicotinamide, respectively. The red dotted arrow highlights key enzyme alterations that underlie decreased NAD^+^ availability during disease conditions. QA: quinolinic acid; QAPRT: quinolinate phosphoribosyltransferase; NAPRT: nicotinate phosphoribosyltransferase; NAAD: nicotinic acid adenine dinucleotide; NADS: NAD synthase; NAMPT: nicotinamide phosphoribosyltransferase; NMNAT: nicotinamide mononucleotide adenylyltransferase; NAMN: nicotinic acid mononucleotide; NMN: nicotinamide mononucleotide; NAM: nicotinamide; NR: nicotinamide ribose; NRK: nicotinamide riboside kinase; LCA: Leber congenital amaurosis; DR: diabetic retinopathy; AMD: age-related macular degeneration; RVO: retinal vein occlusion; TON: traumatic optic neuropathy; NK: neurotrophic keratopathy.

NAD^+^ can be derived from dietary sources via three principal routes: the Preiss-Handler, *de novo*, and salvage pathways (Figure 1) [17, 18]. In mammals, however, the majority of NAD is thought to be generated via the salvage pathway which is controlled by two key enzymes, nicotinamide phosphoribosyltransferase (NAMPT) and nicotinamide mononucleotide adenylyltransferase (NMNAT) [3]. NAMPT converts nicotinamide (NAM) and phosphoribosyl pyrophosphate (PRPP) to nicotinamide mononucleotide (NMN), and NMNAT generates NAD by transferring the adenylyl moiety from ATP to NMN [19, 20]. NMN can also be generated from nicotinamide ribose (NR) when phosphorylated by nicotinamide riboside kinase (NRK). In the final step of NAD biosynthesis, NMN is adenylylated by nicotinamide mononucleotide adenylyltransferase (NMNAT) to form NAD^+^. The salvage pathway is referred to as the salvage pathway because not only does it take advantage of multiple dietary substrates for NAD synthesis but the nicotinamide that is generated as a consequence of NAD^+^ utilization (e.g., by PARP and sirtuin as a substrate for ADP-ribosylation and deacetylation activities, respectively [9, 21] or by the NAD glycohydrolases, CD38 and CD157, which also consume a relatively small percentage of NAD for their activities [5, 22]) is also recouped and recycled to generate more NAD by NAMPT. The significance of the salvage pathway, specifically NAMPT, to retinal health is highlighted by a 2015 report by Kaja et al. [23] that showed that alterations in circulating levels of NAMPT correlate strongly with risk for retinal vein occlusions, conditions in which both ischemia and metabolic disruption are common. Subsequently, Lin et al. [24] confirmed the functional relevance of NAMPT to retinal function by demonstrating that the photoreceptor-specific deletion of this enzyme leads to culminate in retinal degeneration. Recent work in our labs [25] (Thounaojam et al., unpublished work) have similarly demonstrated the importance of this enzyme and the related availability of NAD^+^ also to human retinal pigment epithelial (RPE) and endothelial cells, retinal cell types in which an early senescent phenotype is promoted in the face of inhibition of NAMPT expression and activity and related deficits in NAD^+^ bioavailability. Thus, while there are multiple avenues to NAD^+^ generation, in the ocular environment, involving NAMPT appears to be of paramount importance.

## 3. Approaches to Increase NAD^+^ Levels in Humans

The potential therapeutic utility of NAD^+^ supplementation was demonstrated as early as 1937 when it was demonstrated that it could cure the black tongue in the canine and successfully treat pellagra in the human [26–28]. Accordingly, more recent studies have shown that supplementation with NAD^+^ precursors improves NAD^+^ levels in aged tissues and thereby protects against aging and the development and progression of aging-related diseases [14, 29–33]. Indeed, boosting NAD^+^ metabolism has been shown to extend the lifespan of various organisms, such as yeast, worms, flies, and rodents [11]. A study by Belenky et al. (2007) showed that nicotinamide ribose (NR) supplementation could extend the replicative lifespan of wild-type yeast by more than ten generations [34]. Similarly, in *C. elegans*, NR supplementation extended the average lifespan of wild-type worms [35]. Remarkably, NR has been shown to improve the C57BL/6J mouse lifespan by 5% [36]. Collectively, these data suggest that NAD^+^ replenishment delays normal aging in laboratory animal models.

Different approaches such as modifying diet and exercise and supplementation with various NAD^+^ precursors have found to be clinically relevant in comparison to approaches to reduce NAD^+^ utilization. Herein, we discuss various clinical studies that are conducted using these approaches.

### 3.1. Diet and Exercise

It is well known that daily exercise and caloric restriction boost metabolic health in humans [37]. Exercise in humans influences NAMPT expression; endurance-trained athletes have a twofold higher expression of NAMPT in the skeletal muscle compared with baseline levels in sedentary obese, nonobese, and type 2 diabetic individuals [38]. In another study involving six weeks of endurance exercise, NAMPT protein levels increased in the trained leg only compared to the untrained leg [39]. Also, NAMPT and the subsequent expression of SIRT1 in the adipose tissue of healthy obese participants were found to be increased during a caloric weight loss intervention [40]. A study by Seyssel et al. (2014) provided additional evidence that a state of obesity or overnutrition lowers NAD^+^ levels [41]. Hence, fed versus fasting states influence heavily the NAD^+^/SIRT1 axis. Further studies are required to evaluate the influence of calorie restriction and exercise on retinal NAD^+^ levels and NAMPT expression.

### 3.2. Elevating NAD^+^ Levels via Supplementation with NAD^+^ Precursors

Numerous experimental studies including our own have explored the relevance of enhancing NAD^+^ levels in various cell and tissue types using nicotinamide. Importantly, several clinical trials have demonstrated tolerance and safety of nicotinamide in daily pharmacological doses up to 3.5 g [42–46] and single doses of up to 6 g [47–49]. Very recently, the result of the first clinical trial on NMN was published [50]. The single oral administration of NMN was reported to be safe in healthy men without causing any significant deleterious effects. However, this trial was conducted in only 10 patients receiving a single dose of NMN. Hence, more clinical trials with the larger patient population and longer NMN treatments are required to have a meaningful interpretation of its clinical benefits. In this regard, several ongoing clinical trials (NCT03151239, UMIN000030609, and UMIN000025739) are expected to provide better clinically relevant results.

Nicotinic acid is another NAD^+^ precursor that is well tolerated in humans; however, at high doses, flushing is a major adverse event [51]. The flushing effect is limited when using newly developed synthetic extended- and sustained-release formulations of nicotinic acid; however, exploratory use of nicotinic acid for the purpose of elevating levels of NAD^+^ remains limited [29]. In contrast to nicotinic acid, nicotinamide riboside, another very effective NAD^+^ precursor, does not cause flushing [52]. Indeed, clinical studies demonstrate that daily doses of nicotinamide riboside up to 2000 mg are well tolerated with few side effects and effectively increase NAD^+^ levels by ~60% in peripheral blood mononuclear cells [53–56].

NAD^+^ levels can also be enhanced via dietary means using tryptophan (Trp), an essential amino acid that is metabolized into NAD^+^ through *de novo* biosynthesis in the liver and kidneys. This route is critical for maintaining the NAD^+^ pool, even though the conversion ratio of Trp to NAD^+^ is low in humans, averaging 60 : 1 [57]. Nonetheless, Trp is deemed capable of meeting the metabolic demands of NAD^+^ metabolism in nicotinic acid- and nicotinamide-deficient diets and is well tolerated at high doses, between 30 and 50 mg/kg bodyweight, apart from inducing minor side effects such as drowsiness or sleepiness [58]. To date, however, no dietary supplementation studies are available that assess directly whether boosting NAD^+^ through Trp might be metabolically beneficial in humans.

### 3.3. Reducing NAD^+^ Utilization

With respect to improving NAD^+^ availability to therapeutically enhance mitochondrial function and prevent the bioenergetic crisis that often precipitates cell damage and death in degenerative retinal disease, many have considered raising NAD^+^ levels through exogenous supplementation with NAD^+^ directly or its precursors. However, few have considered the alternate, reducing overall NAD^+^ utilization. As such, no clinical trials with PARP-1 or CD38 inhibitors that focus on improving metabolic variables relevant to the preservation of NAD^+^ have been conducted in humans [29]. This, however, does not imply that this strategy must be abandoned altogether, as a viable work-around to exploit the theoretical metabolic benefit of inhibition of NAD^+^ consumers may present itself in due time, allowing us to assess the efficacy of this strategy in clinical trials.

## 4. Significance of NAD^+^ Metabolism in Ocular Diseases

### 4.1. Leber Congenital Amaurosis 9

Many discussions of the importance of NAD^+^ are related directly to its direct impact on aging and/or the pathogens of age-related disease. This is understandable given that the consequences of altered NAD^+^ metabolism are most often exposed in these conditions. However, while we are aware of the numerous biologic processes that are dependent upon the availability of an adequate supply of NAD^+^, it is important to understand better the significance of NAD^+^ under normal or basal conditions and, therefore, in the absence of aging or related disease. As such, it is quite fitting to start our more detailed discussion of the significance of NAD^+^ metabolism to retinal health and function with Leber congenital amaurosis (LCA), a family of congenital retinal dystrophies that results in severe vision loss at an early age [59]. Leber congenital amaurosis 9 (LCA9) is an autosomal recessive retinal degeneration condition caused specifically by mutations in NMNAT1, a key NAD^+^ biosynthetic enzyme [60] (Figure 1). This was validated by Falk et al. who, using whole-exome sequencing, identified a homozygous missense mutation (c.25G>A, p.Val9Met) in the NAD synthase gene NMNAT1 encoding nicotinamide mononucleotide adenylyltransferase 1 [61]. Around the same time, Koenekoop et al. identified 10 mutant alleles of NMNAT1 in eight families with LCA [62]. Like Falk et al., Koenekoop et al. suggested that the variants would result in altered NMNAT1 structure and function, a hypothesis that these two investigative groups validated via *in vivo* and *in vitro* functional assays. These studies demonstrate convincingly the essential relevance of NAD^+^ not only to the maintenance of retinal structure and function in the adult but importantly also to retinal development and visual function in general. These studies also highlight the sensitivity of retinal neurons in particular to insufficient supplies of NAD^+^, a finding supported also by the more recent work of Lin et al. [24] and Kuribayashi et al. [63] which confirmed the importance of NMNAT1 in retinal development and related photoreceptor health and function.

### 4.2. Glaucoma

Like photoreceptors, the health of retinal ganglion cells (RGCs), cells that serve as a direct liaison between the retina and brain, is too heavily dependent upon NAD^+^. This is evidenced in glaucoma, a complex and multifactorial disease characterized by the progressive dysfunction and loss of RGCs [64]. Major risk factors for glaucoma are increased intraocular pressure (IOP) and age [65]. During aging, the optic nerve, which is formed by the bundled axons of the RGCs, becomes more prone to damage caused by elevated IOP [66]. Indeed, axonal degeneration of RGCs is a hallmark of glaucoma, and studies focused on identifying the mechanisms responsible for the progressive degeneration of RGC axons and how to prevent it are of pivotal importance in the field of glaucoma [67]. Related to axonal health, NMNAT, specifically isoform 1, has been shown to play crucial roles in axonal protection [68, 69]. NMNAT1 is one of three mammalian NAD synthase isoforms whose whole coding sequence has been described as part of the chimeric protein Wallerian degeneration slow allele (Wlds) that is fused to the amino (N)-terminal 70-amino acid fragment of a ubiquitin-protein [70]. Wlds has been reported to be functionally protective with respect to axonal health, and this axon-protective phenotype in the peripheral nervous system has been linked to enhanced expression of the Wlds protein component of NMNAT1 [70]. More specifically, Zhu et al. [67] evaluated the involvement of NMNAT1 and its subcellular localization to the cytoplasm in both acute and chronic RGC disease models. Their study using cytNMNAT1-Tg mice showed that cytoplasmic overexpression of NMNAT1 significantly protects RGC (both axons and soma) from ischemic and glaucomatous injury. Collectively, these findings signify that cytoplasmic overexpression of NMNAT1 can provide RGC's pancellular defense against both acute and chronic retinal injuries. Additionally, this study highlighted the therapeutic importance of nonnuclear NMNAT1 expression in protecting RGCs.

Other mammalian NMNAT isoforms such as NMNAT2 (located in the Golgi apparatus and cytosol) and NMNAT3 (located in the mitochondria) are also reported to exert axonal protection in different neuronal models [71]. Kitaoka and colleagues evaluated the protective effect of NMNAT3 overexpression on optic nerve axonal protection in two different mouse models of glaucoma (the TNF injection model and the hypertensive glaucoma model). Overexpression of NMNAT3 exerted axonal protection against both TNF-induced and IOP elevation-induced optic nerve degeneration. Further, it was reported that the overexpression of NMNAT3 can alter the autophagy machinery, and NMNAT3 may be involved in decreased p62 and increased LC3-II levels in optic nerve degeneration [72]. The above studies have reported on the link between alterations in isoforms of NMNAT, a major player with respect to the biosynthesis of NAD^+^, and axonal health. However, the direct relevance of NAD^+^ itself to aging and/or glaucomatous retina was illustrated directly by Williams et al. who confirmed significant reductions in retinal NAD^+^ content both in normal aging and in glaucoma-prone mice (DBA/2J) [73]. Although the death of RGCs in these models was not indisputably proven to be attributed solely to the reduced availability of NAD^+^, the investigators were able to conclude with confidence that the differences in gene expression and total NAD^+^ levels did impact RGC function. Further, they hypothesized that the age-dependent decline in NAD^+^ levels and subsequent dysregulated energy metabolism, when combined with the stress of elevated intraocular pressure, could have a deleterious impact on mitochondrial function and thereby contribute to the loss of RGCs. The validity of this hypothesis was confirmed by the dose-dependent protection against structural and functional retinal ganglion cell damage conferred in association with dietary supplementation of nicotinamide. Additionally, in the same mouse model, intravitreal injection of an adenoviral vector (AAV2.2) to facilitate overexpression of NMNAT1 also provided significant protection against the development and progression of a glaucomatous phenotype. When used in combination, gene therapy plus nicotinamide treatment, the benefit exceeded that derived from the use of either strategy alone. In another study, Williams et al. demonstrated that Wlds, a protein already shown to be axonal protective, increases retinal NAD^+^ levels [74]. When combined with nicotinamide treatment, Wlds was found to significantly protect mice from glaucomatous neurodegeneration, more than Wlds or nicotinamide alone. Importantly, nicotinamide and Wlds protected somal, synaptic, and axonal compartments, prevented the loss of anterograde axoplasmic transport, and protected animals from visual impairment, a finding demonstrated to be true in 94% of the mouse eyes that were studied [74]. In a follow-up study, Williams et al. provided additional evidence for nicotinamide-mediated protection by including the results from axon counting and optic nerve head analyses [75]. They also show analyses of age- and intraocular pressure-dependent changes in transcripts of NAD-producing enzymes within retinal ganglion cells and that nicotinamide treatment prevents these transcriptomic changes. These data demonstrate that nicotinamide-treated nerves that show no nerve damage are as healthy as nonglaucomatous age-matched controls in terms of their cross-sectional area, axon number, and general morphology, without obvious glial changes. Nicotinamide-treated eyes were also protected from the remodeling and atrophy of the optic nerve head that produces optic nerve cupping, a characteristic feature of human glaucoma. These findings extend previous studies implicating mitochondria in glaucoma by showing that mitochondrial dysfunction is among the first glaucoma-initiating changes within RGCs and that NAD-boosting therapy is potently protective [75]. Collectively, these experiments emphasize the importance of NAD^+^ to the health and function of retinal neurons and additionally suggest that improving mitochondrial health by enhancing the energy metabolism of RGCs through genetic and/or therapeutic augmentation of NAD^+^ could be of benefit in glaucoma [73].

## 5. NAD^+^ and Aging Retina

Although not completely understood with respect to underlying mechanisms, the relevance of NAD^+^ metabolism to aging and longevity has been known for decades. Recent studies have brought to the forefront once again the importance of NAD^+^ metabolism in these processes. The age-dependent decline in NAD^+^ levels has been demonstrated to occur in animal models and humans, in multiple organs including the brain, liver, muscle, pancreas, adipose tissue, and skin [6, 14, 76, 77]. Our recent work coupled with that of others has added the retina, specifically photoreceptor, ganglion, endothelial, and retinal pigment epithelial (RPE) cells, to this growing list of tissues/cell types [24, 25, 67, 68, 72]. Not only do NAD^+^ levels decline with increased age but also an extensive metabolic study conducted by Wang et al., using the cornea, RPE/choroid, and lens, confirmed significant difference in metabolites present within aged (73 weeks old) mouse tissues compared to those present in mice of a relatively young age (6 weeks old) [78]. Together, these data suggest that there is a universal age-dependent decrease of cellular NAD^+^ across species and that most if not all tissues and cell types, excluding possibly only those cells in which mitochondria are absent (e.g., red blood cells), are impacted, to some degree, by the consequent metabolic alterations that emanate. The decrease in NAD^+^ levels is attributed to an imbalance between NAD^+^ synthesis and consumption given that the expression and activity of enzymes critical to NAD^+^ synthesis decline with increasing age despite the fact that the obligatory requirement for NAD^+^ remains high.

### 5.1. Age-Related Macular Degeneration

Age-related macular degeneration (AMD) is a complex multifactorial disease, and as its name implies, age is a primary risk factor for its development [79–81]. Retinal pigment epithelial (RPE) cells and photoreceptor cells, cell types that as discussed above are critically dependent upon the adequate availability of and proper metabolism of NAD^+^, are the principal retinal cell types affected in AMD [82–84]. Congruent with this, studies from our laboratory and others have provided a wealth of evidence in support of the critical importance of NAD^+^ to RPE and photoreceptor health in aging and AMD.

Bai and Sheline [85] showed that in the rodent model of light-induced retinal damage, a model used commonly to model processes relevant to disease development in AMD, retinal NAD^+^ content is significantly reduced. Similarly, NAD^+^ levels in *ex vivo* primary retinal cultures were significantly reduced in association with increased exposure to oxidative stress. Supportive of the prominent causative role that reductions in NAD^+^ have on the propagation of retinal damage in these experimental model systems, nicotinamide injection restored NAD^+^ levels and attenuated light-induced damage to RPE and photoreceptors. The same benefit was attainable via cytoplasmic overexpression of the key biosynthetic enzyme NMNAT1. This study provided pioneering evidence of critical relevance of NAD^+^ to outer retinal health and the potential that therapeutic strategies to restore or augment NAD^+^ might have in treating degenerative retinal diseases such as AMD in which NAD^+^ depletion and consequent damage to photoreceptor cells and RPE are paramount. This finding is supported strongly by several additional studies such as that by Zhu et al. which not only demonstrated the benefit of enhancing NAD^+^ levels in retina/RPE but also further provided insight into the underlying mechanisms to explain this effect: decreased intracellular ROS, inhibition of PARP-1, and upregulated autophagy in RPE cells exposed to prooxidant stimuli [86]. The relevance of NAD^+^ metabolism to AMD is perhaps even more directly demonstrated by a recent report [87], which, using human-induced pluripotent stem cell-derived RPE (hiPSC-RPE) cells, prepared from donors with and without AMD, showed the benefit of the NAD^+^ precursor, nicotinamide, in limiting the expression of key complement and inflammatory proteins linked directly to drusen development and AMD.

**Figure 2:**
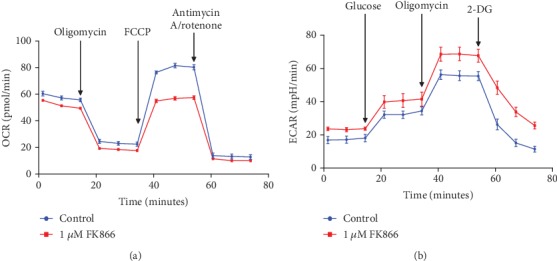
Inhibition of NAMPT activity decreases oxidative phosphorylation and increases glycolysis in human retinal pigment epithelial cells. Human retinal pigment epithelial cells were treated with 1 *μ*M FK866 for 72 hours, and change in oxidative phosphorylation and glycolysis was evaluated using the Seahorse extracellular flux (XF) analyzer. Results are expressed as mean ± SEM for *n* = 6 independent replicates. FCCP: carbonyl cyanide-4-phenylhydrazone; 2-DG: 2-deoxy-d-glucose.

**Figure 3:**
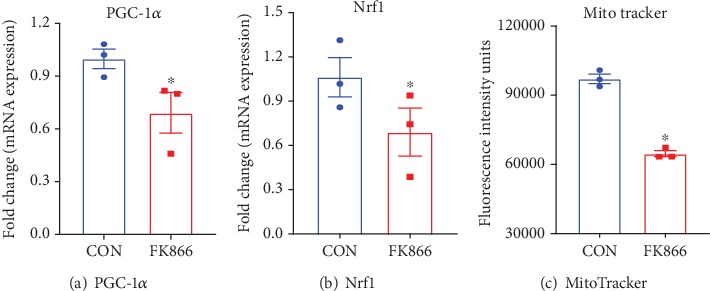
Inhibition of NAMPT activity affects mitochondrial biogenesis in human retinal pigment epithelial cells. Human retinal pigment epithelial cells were treated with 1 *μ*M FK866 for 72 hours, and change in mRNA expression of PGC1a and Nrf1 and density of healthy respiring mitochondria (MitoTracker Green positive) were determined using qPCR and FACS analysis, respectively. Results are expressed as mean ± SEM for *n* = 3 independent experiments. ^∗^*p* < 0.05 vs. control. PGC-1*α*: peroxisome proliferator-activated receptor gamma coactivator 1-alpha; Nrf1: nuclear respiratory factor 1.

In a recent study conducted in our laboratory [25], we too observed a decline in NAD^+^ levels in aged mouse retinas. When searching for the mechanism to explain this phenomenon, we found interestingly that the expression of major contributors in the *de novo* pathway of NAD^+^ biosynthesis was unchanged; however, NAMPT expression declined significantly with age and in RPE specifically. The importance of NAMPT expression and activity was further demonstrated in subsequent *in vitro* and *in vivo* studies using the NAMPT inhibitor FK866. NAMPT inhibition promoted RPE senescence, an effect that was prevented by the exogenous provision of nicotinamide. Thus, while a number of prior studies have demonstrated the impact of modulating NMNAT1 expression on retinal health and function, work by Lin et al. [24] coupled with that of our own implicates NAMPT as a key rate-limiting enzyme in the determination of NAD^+^ availability and the consequent health and function of cells in the outer retina, namely, RPE and photoreceptors. Further evidence for the importance of the NAMPT-mediated NAD^+^ synthesis pathway in the retina comes from the cancer field. NAMPT is overexpressed in many different types of tumors, and its expression appears to be associated with cancer progression [22, 88]. Thus, many cancer biologists have explored the strategy of inhibiting NAMPT expression as a means of reducing tumor growth [88–90]. Although preclinical studies have shown this approach to be very beneficial for reducing various tumor types, this anticancer benefit is associated, unfortunately, with severe retinal toxicity. Additional studies conducted in zebrafish and rodents subjected to NAMPT inhibition have similarly reported severe retinal damage, defined as disruption of architecture and cellular degeneration and loss of cellular layers, vacuolation and thinning of noncellular layers, and, with time and severity, a reactive mononuclear cell infiltrate (interpreted as glial cells) and fibroplasia [91, 92]. Indeed, suppressing NAMPT precipitates a pathologic state that involves photoreceptor and outer nuclear layers and, with progressed severity, the RPE, outer plexiform, and inner nuclear layers. Hence, approaches such as the coadministration of nicotinic acid along with NAMPT inhibitors have been proposed to reduce the incidence of retinal toxicity associated with anticancer NAMPT-inhibiting therapies [93]. Collectively, these studies from an unrelated field to vision science provide further evidence that maintenance of adequate NAD^+^ levels is pivotal for overall retinal health and provide additional strong support of the rationale for targeting NAMPT expression and activity in the retina and, thereby, NAD^+^ metabolism therapeutically to prevent and treat AMD. There is a burgeoning literature in support of the theory that mitochondrial dysfunction contributes significantly to the onset of senescence [94–96]. Hence, building on the information above and our prior report on the significance of the age-associated decline in NAD^+^ levels in RPE senescence, we investigated the role of NAD^+^ in maintaining mitochondrial energy metabolism in RPE cells. As shown in Figure 2, inhibition of NAMPT activity in primary human retinal pigment epithelial (HRPE) cells resulted in a significant decline in oxidative phosphorylation. Alternately, glycolytic respiration was upregulated in NAD^+^-depleted HRPE cells. These observations were a bit surprising as they are in contrast to the findings reported by others using photoreceptor cells wherein FK866 treatment-induced NAD^+^ depletion results in significant impairment in both oxidative phosphorylation and glycolysis [24]. However, these observations highlight the contrasting capabilities of photoreceptors and RPE to handle impairments in energy metabolism. Neuronal cell types (e.g., photoreceptors) appear to be more sensitive to NAD^+^ depletion as it may result in the death of these cells; in contrast, in RPE, cell viability is preserved in lieu of the development of a senescent phenotype. Interestingly, the switch in energy metabolism in RPE from oxidative phosphorylation to glycolysis under stress conditions, though different from energy metabolism in photoreceptor cells subjected to the same conditions, is in accordance with previous studies by others in RPE [97]. We found additionally that NAD^+^ depletion also induced significant downregulation in the expression of the mitochondrial biogenesis markers Nrf1, PGC-1*α*, and TFAM (Figure 3). Of interest, in a hypothesis article, Wei et al. (2019) proposed that NAD^+^ supplementation may restore RPE metabolic dysfunction by inducing mitophagy in AMD [98]. Collectively, these data and recent reports by others and us suggest that NAD^+^ levels are important for maintaining a healthy state of RPE cells, a characteristic that may directly or indirectly impact photoreceptor cell health and function given the major supportive role of RPE relative to this cell type, factors that are highly relevant to the prevention of age-related RPE dysfunction.

Up to this point, our discussion regarding the relevance of NAD^+^ metabolism to AMD has focused largely on RPE. Again, however, photoreceptor cells are too majorly impacted in this disease. The importance of NAD^+^ metabolism to photoreceptor health and function was readily apparent as it relates to the function of the development and function of these cells in Leber congenital amaurosis, a congenital and therefore nonage-associated disease. There is considerable evidence also of the dysfunction of these cells in the aging retina and/or AMD congruent with the decline of NAD^+^ under these conditions. Studies performed by a research group at Washington University School of Medicine, USA, demonstrated that nicotinamide supplementation (100 and 300 mg/kg/day NMN doses) prevented deficits in rod cell function as ascertained by electroretinography studies in aged mice [99]. The critical relevance of NAD^+^ metabolism in photoreceptor cells was additionally demonstrated in mice in which NAMPT was deleted specifically in rod and/or cone photoreceptors. Rod-specific deletion of NAMPT in mice significantly impaired impairs photoreceptor survival and vision, whereas NAMPT deletion in cone photoreceptors of mice significantly decreased photopic visual acuity, phenotypes that the authors demonstrated to be linked strongly to altered function of the mitochondrial deacetylase SIRT3 consequent to NAD^+^ deficiency [24]. Thus, it appears that augmenting NAD^+^ availability whether through exogenous supplementation or genetic modifications that promote endogenous biosynthesis may be of benefit in protecting photoreceptors and RPE thereby preserving visual health and function in aging and/or diseased (AMD) retina [24, 100].

### 5.2. The Relevance of NAD^+^ Metabolism in Other Eye Diseases

In addition, to the major sight-threatening retinal diseases mentioned previously, alterations in NAD^+^ metabolism have been reported to occur also in association with other retinal and nonretinal ocular diseases including diabetic retinopathy [101], retinal vein occlusion [23], neurotrophic keratopathy [102], and traumatic optic neuropathy [103]. A brief mention of current information available relevant to these ocular disorders is provided below. Retinal vascular occlusions (RVOs) are prevalent in about 0.5% of the population. However, the incidence of RVOs increases with increasing age though variable with ethnicity and/or race. Indeed, retinal vein occlusions are a common cause of retinal vascular disease, second only to diabetic retinopathy, a leading cause of blindness worldwide. About half of the cases of RVOs occur in patients older than 65 years of age, and more than half of these cases are found in patients with cardiovascular diseases. Interestingly, reduced serum levels of NAMPT have been reported to be associated inversely with the incidence and severity of RVOs [23]. However, little detailed confirmatory information is available. As such, this clinical observation should be further evaluated in detail to better undertint the role of NAMPT in RVOs.

RGC injury is an important pathological feature in the pathological process of diabetic retinopathy. Hence, protecting RGCs from high glucose-induced injury is a promising strategy that improves neuronal damage in diabetic retinopathy. Zhou et al. evaluated the role of NMNAT1, in high glucose-induced RGC injury [101]. The data in this study show that NMNAT1 knockdown could aggravate RGC injury and accelerate the development of RGC apoptosis in response to high glucose stress. Additionally, our recent unpublished work highlights the importance of NAD^+^ metabolism to retinal endothelial cells, photoreceptors, and RPE, cell types too affected robustly in the diabetic retina. NAD^+^ metabolism in aging and therefore age-related diseases such as AMD has received much attention; however, additional effort to understand the importance of NAD^+^ metabolism in the diabetic retina is also warranted.

Recent work suggests that NAD^+^ metabolism may also impact the susceptibility of retinal cells to traumatic injury. Therefore, augmentation of NAD^+^ availability may be of therapeutic relevance also in this ocular condition. Supportive of this, it has been shown that P7C3, a member of the aminopropyl carbazole class, activates NAMPT contributing to increased NAD^+^ generation [104]. This is supported by a study from the research group of Dr. Hidehiro Oku that evaluated the protective effects of P7C3 on optic nerve injury and found that 5 mg/kg/day of P7C3-A20 for 3 days had a neuroprotective effect on RGCs after the optic nerve crush in rats [103].

The impact of NAD^+^, particularly reductions in its availability, certainly extends beyond the retina. Neurotrophic keratopathy is a degenerative disease characterized by impaired corneal epithelium healing and the absence of corneal sensitivity that renders the cornea vulnerable to injury [105]. The incidence of neurotrophic keratitis increases with age. To determine how corneal epithelial denervation induces apoptosis, Li et al. [102] studied the significance of NAD^+^ levels and NAMPT expression in a mouse model of corneal denervation. The results showed that the denervation decreased epithelial NAD^+^ content by reducing NAMPT expression resulting in corneal epithelial apoptosis. Further, the inhibition of NAD biosynthesis by the NAMPT inhibitor recapitulated corneal denervation-induced epithelial detachment and cell apoptosis, which was partially improved by the replenishment of NAD^+^ or NMN. In conclusion, this study demonstrated that corneal denervation which impaired the epithelial NAD^+^ level caused apoptosis and epithelial detachment, suggesting that corneal innervations may control epithelial homeostasis by regulating NAD^+^ biosynthesis. This phenomenon is worthy also of additional exploration.

## 6. Concluding Remarks

NAD^+^ is a key cofactor required for the propagations of a plethora of reactions of metabolic importance. Therefore, is not surprising that when levels of NAD^+^ levels are compromised, the consequences can be and often are quite severe as we have discussed in aging and/or diseased retina. NAD^+^ is certainly a critical regulator of aging and longevity and, therefore, a promising target for the development of therapies aimed a treating aging and aging-associated diseases. Thus, any discussion of the impact of mitochondrial oxidative stress and altered energy metabolism to aging and longevity would be incomplete without considering factors relevant to the biosynthesis and utilization of NAD^+^, hence, the basis of this review. Numerous studies using mouse models have demonstrated the beneficial effects of NAD precursors against metabolic disorders, cancer, and neurological disorders. Additionally, several studies have clearly shown that genetic or nutritional activation of NAD metabolism promotes lifespan extension in various organisms including mammals. However, comparatively few studies have investigated the role of NAD^+^ metabolism in the regulation of visual health and function. Based on the available literature, it can be surmised that while the salvage pathway for NAD^+^ synthesis is significantly altered in retinal aging and various conditions of retinal degeneration, the *de novo* pathway seems to be unaffected. Moreover, there seem to be cell type-dependent differences in the affected enzymes. For example, while NAMPT expression is more important to outer retinal cells (RPE and photoreceptors), NMNAT plays a significant role in maintaining the health and function of RGC cells, neurons of the inner retina. Overall, various strategies aimed at improving retinal NAD^+^ metabolism have shown some promising results in improving visual function in laboratory animals. Thus, similar detailed studies in humans are warranted. Collectively, we hope that the present review will confirm the importance of NAD^+^ metabolism in the aging retina and other retinal diseases and lead to further clinical studies on evaluating the role of NAD supplementation in improving vision.
